# Advances in Pd Nanoparticle Size Decoration of Mesoporous Carbon Spheres for Energy Application

**DOI:** 10.1186/s11671-015-1113-y

**Published:** 2015-10-30

**Authors:** Beata Zielinska, Beata Michalkiewicz, Ewa Mijowska, Ryszard Józef Kalenczuk

**Affiliations:** Institute of Chemical and Environment Engineering, West Pomeranian University of Technology, Szczecin, Pułaskiego 10, 70-322 Szczecin, Poland

**Keywords:** Hydrogen storage, Carbon sorbents, Mesoporous carbons, Pd nanoparticles

## Abstract

Pd nanoparticles with different sizes and diameter distributions were successfully deposited on the surface of disordered mesoporous carbon spheres (DMHCS). The size and diameter distribution of the Pd particles were controlled by the application of different experimental conditions. Two methods of synthesis (reflux and impregnation) and two Pd precursors (palladium (II) acetyloacetonate (Pd*(*acac*)*_2_) and palladium (II) acetate (Pd(OAc)_2_)) were investigated and compared for the preparation of Pd-decorated DMHCS. The hydrogen storage properties of the pristine DMHCS and Pd-modified DMHCS at 40 °C and a pressure range of 0–45 bar were studied. The results showed that Pd-supported carbon samples synthesized in the presence of Pd(OAc)_2_ exhibited enhanced hydrogen storage capacity in respect to the pristine DMHCS. The maximum hydrogen storage of 0.38 wt.% exhibited the sample with the Pd nanoparticle diameter distribution of 2–14 nm and the average Pd crystallite size of 7.6 nm. It was found that the Pd nanoparticle content, size, and diameter distribution have a noticeable influence on H_2_ storage capacity.

## Background

In the past few decades, a variety of energy storage and conversion materials have been applied to high-powered energy devices such as lithium batteries, supercapacitors, fuel cell, and solar energy cells [1–3]. In comparison to the conventional energy materials, carbon nanomaterials have attracted extensive attention because of their unusual size and surface dependent properties useful in enhancing energy conversion and storage performance [2]. In particular, the mesoporous carbons have been extensively studied due to their potential applications in hydrogen storage [1, 4].

There have been many studies of hydrogen adsorption which showed that H_2_ storage capacity is enhanced by added metal particles (Ag, Pd, Pt, Ri, Fe, Ni, and Ru) to carbon materials [5–18]. This phenomenon is well known for heterogeneous catalysis and called as hydrogen spillover [6, 7]. Hydrogen storage properties of different carbon materials functionalized with metal particles such as activated carbon, carbon nanotubes, carbon nanofibers, and graphene have been extensively studied. For example, B. P. Vinayan et al. [8] studied the hydrogen storage properties of nitrogen-doped graphene and Pd-decorated nitrogen-doped graphene. The results showed that hydrogen uptake capacity of nitrogen-doped graphene and palladium-decorated nitrogen-doped graphene (32 bar and 25 °C) is 0.42 and 1.25 wt.%, respectively. Moreover, the authors argued that high dispersion of Pd nanoparticles on nitrogen-doped graphene nanoplatelets and strong adhesion between metal and graphene enhanced the spillover mechanism. The effect of nickel distribution and content in Ni-modified carbon nanospheres on hydrogen storage capacity have been investigated [9]. It was found that Ni distribution has a noticeable influence on H_2_ storage capacity. The samples with the most homogeneous Ni distribution exhibited the highest hydrogen uptake capacity. Moreover, the authors stated that storage properties of Ni-doped carbon spheres were influenced by the amount of nickel. Pd nanoparticles with different sizes and diameter distributions were deposited on the surface of carbon nanotubes and synthesized using a simple in situ technique (sonication and stirring) [10]. The authors revealed that the system composed of carbon nanotubes and Pd nanoparticles has a potential as hydrogen storage medium due to the enhanced H_2_ adsorption capacity and stability after multiple adsorption–desorption cycles. Moreover, the hydrogen storage properties of Pd supported in hollow carbon spheres (HCS) under room temperature and at partial pressures were investigated [11]. It was found that the Pd-decorated HCS sample with suitable diameters of Pd nanoparticles was more favorable for H_2_ storage, even when lower mass of Pd was used. The maximum hydrogen uptake capacity (0.36 wt.%) showed the material with the Pd nanoparticle diameter of 11 nm, and the hydrogen storage capacity was enhanced by the factor of two in respect to the pristine HCS.

The aim of this work is to examine the effects of palladium source and synthesis procedure on the optical, morphological, nanoparticles size distribution, and hydrogen adsorption capacity of DMHCS. In the study, carbon spheres were produced via chemical vapor deposition (CVD) method using disordered mesoporous silica templates (SiO_2_@m-SiO_2__C18TMS) and ethylene as a carbon source. Two methods of synthesis such as reflux and impregnation and two Pd precursors (palladium (II) acetyloacetonate and palladium (II) acetate) were selected and compared for the preparation of Pd-modified DMHCS. Finally, the hydrogen storage capacity of the materials was revealed.

## Methods

### Synthesis of SiO_2_@m-SiO_2__C18TMS Template

The disordered mesoporous hollow silica sphere templates (SiO_2_@m-SiO_2__C18TMS) were synthesized by using octadecyltrimethoxysilane (C18TMS) as the surfactant and tetraethyl orthosilicate (TEOS) as the silica source. TEOS (3 cm^3^) was added to a mixture of ethanol (50 cm^3^), concentrated ammonia (28 wt %, 4 cm^3^), and deionized water (4 cm^3^). The obtained mixture was stirred for 24 h, and then the solution of C18TMS (1 cm^3^) and TEOS (2.5 cm^3^) was added dropwise while stirring. The solution was sonicated and stirred for another 6 h. The final product was isolated by filtration, cleaned with ethanol and water several times, and dried in air at 100 °C for 24 h.

### Synthesis of DMHCS

The procedure for the preparation of the mesoporous hollow carbon spheres was in accordance with the reported method by Wenelska et al. [11]. In a typical synthesis, SiO_2_@m-SiO_2__C18TMS template was placed in an alumina boat and set in a tube furnace. Argon and ethylene were introduced in a flow rate of 100 and 30 sccm, respectively. The temperature was raised to 800 °C, and the chemical vapor deposition reaction time was 3 h. Afterwards, the furnace was cooled to room temperature in Ar. Next, the produced SiO_2_@m-SiO_2__C spheres were treated with hydrofluoric acid. Here, the HF treatment step was repeated twice to remove all silica templates from carbon spheres. After this step, the obtained mixture was poured into water (5 dm^3^). Finally, the sediment was recovered by filtration, washed several times with distilled water, and dried in air at 100 °C for 24 h. The resulting product, prepared in the abovementioned way, is labeled as DMHCS.

### Synthesis of Pd-Modified DMHCS

Two palladium sources (palladium (II) acetyloacetonate Pd*(*acac*)*_2_ and palladium (II) acetate Pd(OAc)_2_) and two synthesis procedures (reflux and impregnation) were used for the preparation of Pd nanoparticle-decorated DMHCS. In the procedure with reflux, the mixture of DMHCS (50 mg), palladium (II) acetyloacetonate or palladium (II) acetate (50 mg), and ethanol (150 cm^3^) was placed in a flask fitted with a condenser and refluxed at 110 °C for 24 h. In the case of an impregnation technique, 50 mg of DMHCS and 50 mg of palladium (II) acetyloacetonate or palladium (II) acetate were dispersed in 150 cm^3^ of ethanol and stirred at the room temperature for 12 h. Finally, each sample was filtered, washed with ethanol and distilled water, and dried in air at 100 °C for 24 h. The samples synthesized in the abovementioned ways are labeled as Pd-R1, Pd-R2, Pd-I1, and Pd-I2 (R reflux, I impregnation, 1 palladium (II) acetyloacetonate, and 2 palladium (II) acetate).

### Characterization

High transmission electron microscopy (TEM) has been used to examine the structural details of the synthesized samples (Tecnai F30 with a field emission gun operating at 200 kV). The crystallographic structures of the prepared samples were confirmed by X-ray diffraction analysis (XPert PRO Philips diffractometer, CuK_α_ radiation). The diffracted intensity of CuK_α_ radiation (*λ* = 1.54 Å, 35 kV, and 30 mA) was measured in a 2*θ* range between 10 and 90. The average Pd crystallite size (*D*) was calculated using the Scherrer equation:

1 $$ D=\frac{K\lambda }{\left(B\hbox{-} b\right) \cos \theta } $$

where *K* is the shape factor (0.9 [17], *λ* is the X-ray wavelength (1.54 Å), *B* is the broadening of the diffraction line measured at half maximum intensity (FWHM), *b* is the line width originating solely from instrumental broadening, and *θ* is the Bragg angle corresponding to the (111) plane. Raman scattering was conducted on a Renishaw micro Raman spectrometer (*λ* = 785 nm). Thermogravimetric analysis (TGA) measurement was carried out in air using the DTA-Q600 SDT TA Instrument with a temperature scan rate of 10 °C per minute. The specific surface area was calculated by the Brunauer-Emmett-Teller (BET) method via Micromeritics ASAP 2010 M instrument. Moreover, hydrogen adsorption capacity was investigated using a Sievert-type volumetric apparatus (IMI Series-Hiden Isochema), which fully automatically measures adsorption and desorption isotherms at 40 °C and up to pressure of 4.5 MPa using ultra high purity hydrogen gas (99.999 %).

## Results and Discussion

**Fig. 1:**
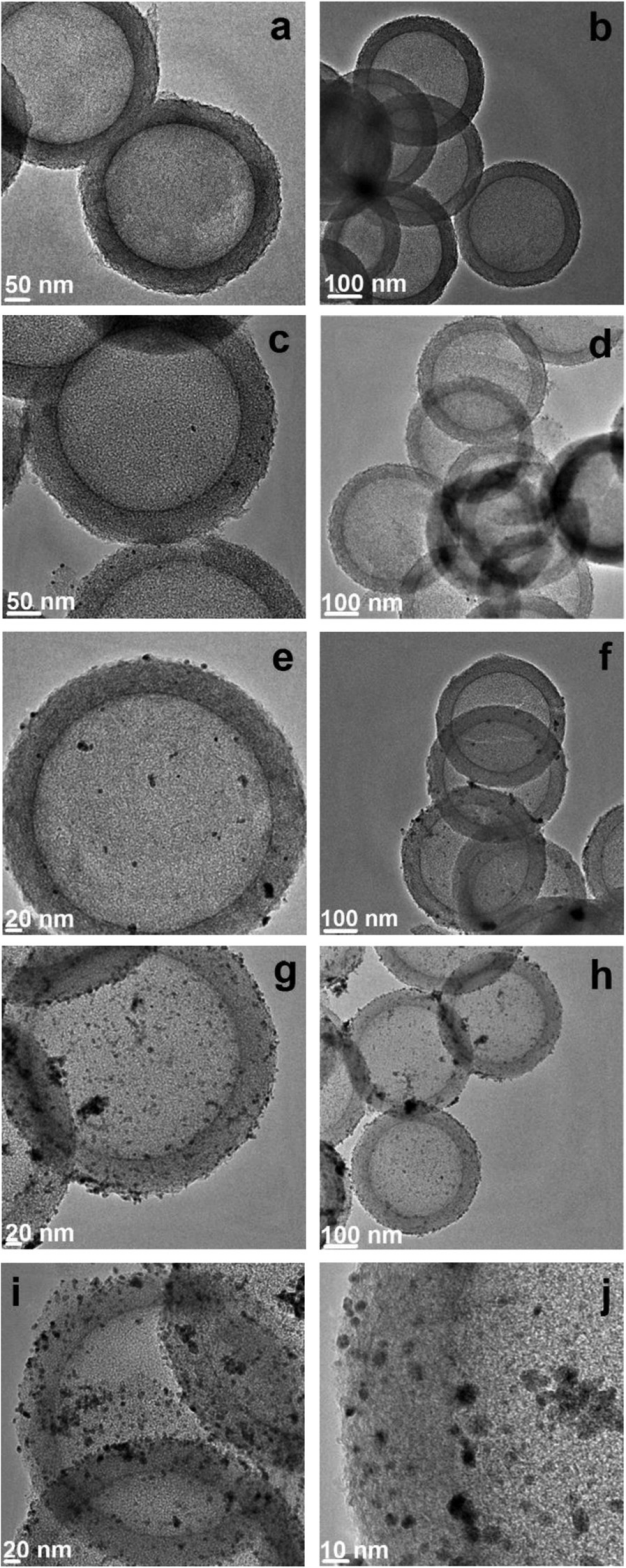
TEM images of the pristine DMHCS (**a**, **b**) and DMHCS deposited with Pd nanoparticles: Pd-I1 (**c**, **d**), Pd-R1 (**e**, **f**), Pd-I2 (**g**, **h**), and Pd-R2 (**j**, **i**)

**Fig. 2:**
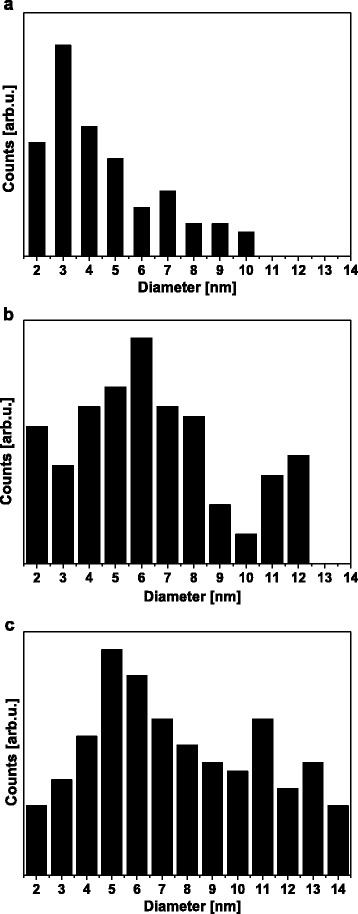
Pd nanoparticle size distributions of **a** Pd-R1, **b** Pd-I2, and **c** Pd-R2

Figure 1 shows representative TEM images of the pristine DMHCS and Pd nanoparticles modified DMHCS samples synthesized by two different procedures (reflux and impregnation) and in the presence of two different palladium sources (Pd*(*acac*)*_2_, and Pd(OAc)_2_). The DMHCS (images a, b) are very uniform in diameter of about 400 nm and the shell thickness is around 50 nm. Figure 1c–f shows TEM images of the Pd-I1 and Pd-R1 samples synthesized by impregnation and reflux techniques, where palladium (II) acetyloacetonate was a source of Pd. For Pd-I1 (Pd*(*acac*)*_2_, impregnation), only a trace amount of Pd nanoparticles were deposited on the surface of DMHCS. It was found that the amount of Pd supported on DMHCS slightly increases when Pd*(*acac*)*_2_ and reflux method were employed (Pd-R1). Moreover, for both synthesis procedures Pd nanoparticles have been successfully deposited on DMHCS when Pd precursor was palladium (II) acetate (Pd(OAc)_2_) (Fig. 1g–j). Figure 2 shows the Pd particle diameter distribution of three Pd-modified samples. In these histograms it is clearly visible that Pd-R1, Pd-I2, and Pd-R2 are composed of Pd particles with the different diameter distribution in the range of 2–10, 2–12, and 2–14 nm, respectively.

**Fig. 3:**
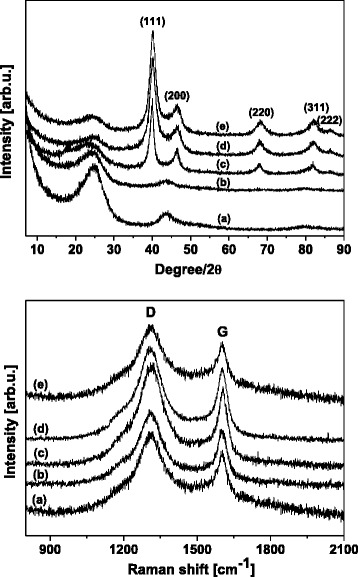
XRD patterns (*upper panel*) and Raman spectra (*bottom panel*) for *a* DMHCS, *b* Pd-I1, *c* Pd-R1, *d* Pd-I2, and *e* Pd-R2

The crystallographic composition of the synthesized samples was studied via X-ray diffraction (XRD). Figure 3 (upper panel) shows the XRD patterns of the pristine DMHCS (pattern a) and Pd nanoparticle-decorated DMHCS (patterns: b Pd-I1, c Pd-R1, d Pd-I2, e Pd-R2). XRD pattern of DMHCS shows two broad diffraction peaks at 2*θ* value of 25° and 45° corresponding to the (002) and (100) planes of graphitic carbon, respectively [18]. No diffraction peaks corresponding to the new phase of palladium are found for Pd-I1. The absence of the reflections for palladium could result from the low concentration of Pd nanoparticles deposited on DMHCS (below the detection limit of XRD). It indicates that using Pd*(*acac*)*_2_ as the precursor together with impregnation method did not induce the deposition of Pd nanoparticles on the surface of DMHCS. For Pd-R1, Pd-I2, and Pd-R2 samples, intense peaks at 2*θ* value of 40°, 46°, 68°, 82°, and 87° are observed. All those reflections are characteristic to the palladium phase (JCPDS card no. 50-0681) and correspond to the (111), (200), (220), (311), and (222) planes, respectively [19]. Here, it is clearly seen that the intensity of the Pd reflections increases in the following order: Pd-R1 > Pd-I2 > Pd-R2, indicating increased crystallinity. The calculated average Pd crystallite size is about 5.3 nm (Pd-R1), 6.4 nm (Pd-I2), and 7.6 nm (Pd-R2). These results are consistent with the TEM analysis of the samples.

The Raman spectra of (a) DMHCS, (b) Pd-I1, (c) Pd-R1, (d) Pd-I2, and (e) Pd-R2 are presented in Fig. 3 (bottom panel). The Raman responses of all studied samples show D and G peaks at about 1311 and 1603 cm^−1^, respectively. The D band corresponds to the presence of amorphous carbon due to the surface defects of carbon nanomaterials and graphitic carbon while the G band is associated with the C–C stretching vibration in graphitic materials [20–22]. The intensity ratio of the D and G bands (*I*_D_/*I*_G_) is commonly known as a parameter to characterize the quality of carbon materials. A high intensity ratio indicates a high degree of disorder in the carbon samples [22, 23]. The calculated values of *I*_D_/*I*_G_ ratio which provide insight regarding the quality of the pristine and modified DMHCS are 1.258, 1.269, 1.283, 1.294, and 1.308, respectively. Comparing the ratios of *I*_D_/*I*_G_ for each sample, it can be argued that all Pd-decorated DMHCS samples have more structural defects than the pristine carbon spheres.

**Fig. 4:**
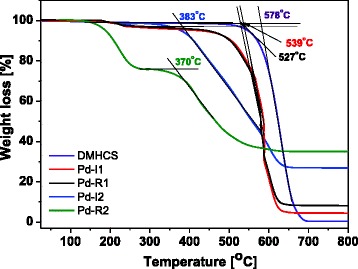
TGA profiles of DMHCS, Pd-I1, Pd-R1, Pd-I2, and Pd-R2

Figure 4 shows the TGA profiles of (a) DMHCS, (b) Pd-I1, (c) Pd-R1, (d) Pd-I2, and (e) Pd-R2. TGA results showed that burning of the pristine DMHCS begins at around 578 °C. Above this temperature, the weight loss rapidly increases until all of DMHCS are exhausted at about 700 °C. The residual weight of DMHCS after the combustion was 0 wt.%. It indicates the high purity of the synthesized DMHCS. Figure 4 indicates that the thermal stability of Pd-modified samples decreased in comparison to the pristine carbon spheres. For Pd-I1, Pd-R1, Pd-I2, and Pd-R2 samples, the burning of DMHCS begins at around 539, 527, 383, and 370 °C, respectively. This may be due to the interaction of Pd nanoparticles with carbon atoms inducing defects in the crystal structure of DMHCS [10]. Moreover, as shown in Fig. 4, Pd-R2 sample loses its weight in two stages. The first stage of weight loss at about 200 °C (~25 wt.%) is due to the decomposition of Pd(OAc)_2_ precursor [24]. The second weight loss (370–630 °C) is attributed to the burning of DMHCS. Moreover, according to the data from TG measurements, it was found that the content of palladium in Pd-I1, Pd-R1, Pd-I2, and Pd-R2 is 4.31, 8.62, 26.64, and 34.95 wt.%, respectively.

**Fig. 5:**
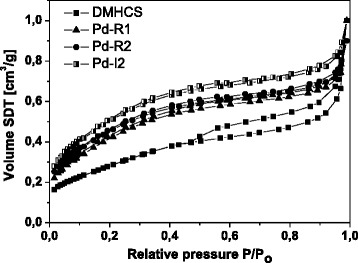
N_2_ adsorption–desorption isotherms for the pristine DMHCS and Pd-modified DMHCS

The N_2_ adsorption/desorption isotherms at 77 K of DMHCS, Pd-R1, Pd-I2, and Pd-R2 are shown in Fig. 5. The isotherms exhibit the type IV curve which indicates the presence of mesoporosity in these samples [25]. The total specific surface area of the pristine DMHCS is 1387 m^2^/g. For Pd-R1, Pd-I2, and Pd-R2 samples, BET surface area significantly decreased to 717, 706, and 569 m^2^/g, respectively. Moreover, the total pore volume of DMHCS (0.737 m^3^/g) is considerably higher than that of Pd-R1 (0.406 m^3^/g), Pd-I2 (0.385 m^3^/g), and Pd-R2 (0.260 m^3^/g). This indicates that Pd nanoparticles block the pores of the carbon spheres which caused a decrease in the surface area and total pore volume of the modified carbons. The characteristics of the samples are presented in Table 1.

**Table 1:** Characteristic of the pristine DMHCS and Pd-decorated DMHCS

Sample	Average Pd crystallite size (nm)	Pd diameter distribution (nm)	BET surface area (m^2^/g)	Total pore volume (cm^3^/g)
DMHCS	–	–	1387	0.737
Pd-R1	5.3	2–10	717	0.406
Pd-I2	6.4	2–12	706	0.385
Pd-R2	7.6	2–14	569	0.260

**Fig. 6:**
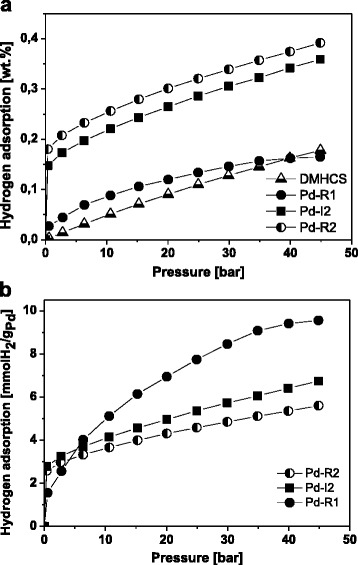
Hydrogen adsorption capacity at 40 °C for DMHCS, Pd-R1, Pd-I2, and Pd-R2 samples: **a** wt.% and **b** per gram of Pd

Finally, the hydrogen storage studies for the produced materials have been revealed. The H_2_ storage properties of the samples were investigated for the pristine DMHCS and only for the most interesting Pd-modified DMHCS that is for the Pd-R1, Pd-I2, and Pd-R2. Only for these samples Pd nanoparticles were successfully deposited on DMHCS. Figure 6 shows the hydrogen sorption isotherms of the samples measured in the pressure range of 0–45 bar and at the temperature of 40 °C. For all studied carbon materials, the maximum capacities have been achieved at 45 bar of hydrogen. Under these experimental conditions (Fig. 6a), the hydrogen uptake capacities were 0.17, 0.16, 0.35, and 0.38 wt.% for DMHCS, Pd-R1, Pd-I2, and Pd-R2, respectively. The sample produced via reflux and in the presence of Pd*(*acac*)*_2_, (Pd-R1) has similar hydrogen storage properties with that of the pristine DMHCS. This can be explained by two effects: (1) only trace amount of Pd nanoparticels were deposited on DMHCS (~9 wt.%) and (2) lower BET surface area with respect to the pristine DMHCS (717 m^2^/g). Pd-I2 and Pd-R2 samples obtained via both studied synthesis procedures and Pd(OAc)_2_ as a source of Pd nanoparticles exhibit higher hydrogen capacity than that of the DMHCS. Hydrogen spillover effect is proposed to explain the increase of the storage capacity of these two Pd-modified samples. First, H_2_ molecules interact with catalyst particles and dissociate into hydrogen atoms. Next, H atoms migrate from the catalyst particles to the receptor material and further diffuse throughout the entire receptor. Here, Pd nanoparticles act as the catalyst for H_2_ dissociation and DMHCS is the receptor [26]. Moreover, the highest hydrogen uptake of 0.38 wt.% exhibited the sample with Pd content of about 35 wt.%, the broadest Pd nanoparticle diameter distribution of 2–14 nm and the highest average Pd crystallite size of 7.6 nm. It indicates that the deposition of Pd nanoparticles on the surface of carbon materials with broad diameter distribution and large particle size and content leads to higher hydrogen storage capacity. The large Pd particles provide more interstitial places for the formation of hydrides which causes that more hydrogen per palladium atom can be absorbed in the bigger particles and hydrogen storage capacity increases. It has been reported that the formation of Pd-H antibonding states exhibited strong particle size dependence [27, 28]. The hydrogen uptake curves of palladium-decorated DMHCS are also normalized by gram of Pd (Fig. 6b). The data presented in Fig. 6b showed that H_2_ uptake capacity of Pd-modified samples decreases with increasing the Pd loading. Here, the hydrogen spillover effect can explain this behavior [29].

From the above described results, it can be concluded that selecting a suitable palladium precursor and preparation route are crucial to obtain Pd nanoparticle-deposited carbon sorbents with high hydrogen capacity. The results indicate that reflux doping method and Pd(OAc)_2_ as a palladium source are more favorable for the preparation of Pd nanoparticle-deposited disordered mesoporous hollow carbon spheres. Moreover, optimization of the Pd nanoparticle content, size, and diameter distribution is essential to improve the hydrogen uptake of carbon sorbents.

## Conclusions

In summary, the hydrogen storage capacity at 40 °C and in the pressure range of 0–45 bar of the pristine and Pd-decorated DMHCS was studied. For the synthesis of modified carbon spheres, the two preparation routes (reflux and impregnation) and the two Pd sources were investigated and compared. The obtained results showed that the synthesis method and Pd nanoparticles precursor had a significant impact on the morphological, optical, thermal, and hydrogen storage properties of palladium-modified DMHCS. Moreover, Pd nanoparticles size, content, and diameter distribution have a significant influence on hydrogen storage uptake. It was found that Pd-deposited DMHCS samples (Pd-I2 and Pd-R2) produced in the presence of palladium (II) acetate exhibited enhanced hydrogen capacity in comparison to the pristine DMHCS. The maximum H_2_ storage (0.38 wt.%) showed Pd-R2 sample obtained via reflux technique.
